# NAC25 transcription factor regulates the degeneration of cytoplasmic membrane integrity and starch biosynthesis in rice endosperm through interacting with MADS29

**DOI:** 10.3389/fpls.2025.1563065

**Published:** 2025-03-18

**Authors:** Rong Li, Ming-Wei Wu, Jinxin Liu, Xintong Xu, Yiqun Bao, Chun-Ming Liu

**Affiliations:** ^1^ College of Life Sciences, Nanjing Agricultural University, Nanjing, China; ^2^ Institute of Crop Sciences, Chinese Academy of Agricultural Sciences, Beijing, China; ^3^ Key Laboratory of Plant Molecular Physiology, Institute of Botany, Chinese Academy of Sciences, Beijing, China; ^4^ College of Life Sciences, University of Chinese Academy of Sciences, Beijing, China; ^5^ School of Advanced Agricultural Sciences, Peking University, Beijing, China

**Keywords:** transcription factor, NAC25, MADS29, starch synthesis, cytoplasmic membrane degeneration, rice grain filling

## Abstract

**Introduction:**

Grain filling is a crucial stage of the rice endosperm development. During this process, the endosperm accumulates abundant storage products such as starch and proteins, which determine both the yield and quality of the grain.

**Methods:**

Here, we analyzed the expression of *NAC25* transcription factor via qRT-PCR and histochemical GUS assays, and obtained its mutants by CRISPR/Cas9-based gene editing in ZH11.

**Results and discussion:**

The results showed that *NAC25* was expressed specifically in developing rice endosperm, and knockout of NAC25 led to delayed degeneration of cytoplasmic membrane integrity, reduced starch accumulation and chalky starchy endosperm. We showed that NAC25 interacted with MADS29, a MADS family transcription factor whose mutant also showed defective grain filling. These results provide novel insight into the transcriptional regulation of rice grain filling.

## 1 Introduction

Rice (*Oryza sativa* L.) is one of the most important staple food crops worldwide and servers as a model plant for molecular genetic research in cereals. Endosperm, which occupies the majority of the mature rice grain, is an important energy source for both human food and seedling growth. The development of endosperm initiates with a double fertilization and culminates in the formation of a dehydrated, firm and semi-transparent grain. This process can be divided into four primary phases: coenocytic nuclear division (0-2 days after pollination, DAP), cellularization (3-5 DAP), the storage product accumulation (6-20 DAP), and finally maturation (21-30 DAP) (Wu et al., 2016; Liu et al., 2022a). The stage of storage product accumulation, which partially overlaps with the differentiation of aleurone and starchy endosperm (6-9 DAP), is often referred to as the grain filling stage and holds immense significance in determining the ultimate grain characteristics and yield (Jongkaewwattana and Geng, 2001). In the rice endosperm, starch accumulates within amyloplasts, which often consist of one to several dozen smaller non-fusing starch granules (Zhao et al., 2018; Yan et al., 2024). These starch granules generally display a polyhedral shape with sharp-edges (Kawagoe, 2013). Starch biosynthesis that involves a series of intricate and coordinated enzymatic reactions has been comprehensively investigated across various plant species (Huang et al., 2021; Seung and Smith, 2019; Cao et al., 2024). In the mature endosperm, the starchy endosperm consists of dead cells resulted from a gradual degeneration of cytoplasmic membrane integrity and programmed cell death (PCD) (Liu et al., 2022a). Metacaspases (MC) and vacuolar processing enzymes (VPE) have been implicated in the PCD process (Huang et al., 2015; He et al., 2023). During this process, an increase in mitochondrial membrane permeability has also been observed (Kobayashi et al., 2013; Teng et al., 2024; Lam et al., 2001). Starch accumulation in the starchy endosperm occurs concurrently with the degeneration of cytoplasmic membrane integrity. Recently, a model has been proposed suggesting that the formation of a large free-trade compartment with shared cytoplasms allow sugars and amino acids to move freely within the starchy endosperm, thereby facilitating effective starch accumulation (Wu et al., 2016; Liu et al., 2022a).

Transcription factors, as pivotal regulators of gene expression, exert a critical role in rice grain filling and starch biosynthesis. RSR1 and SERF1 act as transcriptional repressors, inhibiting starch biosynthesis during the grain filling (Fu and Xue, 2010; Schmidt et al., 2014). Conversely, MADS1, MADS6, bZIP10 and bZIP60 have been identified as positive regulators of that promote starch synthesis (Zhang et al., 2010; Jiang et al., 2024; Yang et al., 2022; Cao et al., 2022; Liu et al., 2023a; Liu et al., 2025b). Additionally, *MADS29*, expressed in nucellar projection region and endosperm of the rice grain, is involved in regulating the PCD of both the maternal and filial tissues, and consequently the rice grain filling (Yin and Xue, 2012; Yang et al., 2012; Nayar et al., 2013). Mutation of *MADS29* resulted in delayed grain filling and a decrease in grain quality (Yang et al., 2012; Nayar et al., 2013). During the process of rice grain filling, different transcription factors may collaborate by assembling into specific complexes. Examples of these complexes include OsBP-5-OsEBP-89, RISBZI/bZIP58-RPBF, OsNF-YB9-SPK, NF-YB1-YC12-bHLH144, NF-YB1-ERF115, NF-YB1-MADS14, NF-YB1-MYB73, and MADS1-NF-YB1-YC12 (Zhu et al., 2003; Yamamoto et al., 2006; Kawakatsu et al., 2009; Niu et al., 2021; Bello et al., 2019; Xu et al., 2016; Feng et al., 2022; Liu et al., 2024, Liu et al., 2025b).

Plant-specific NAC genes represent a major family of transcription factors for their roles in plant growth, development, and responses to both abiotic and biotic stresses (Han et al., 2023). Several NAC family transcription factors showed specific or elevated expression in the caryopsis, and are involved in regulating grain filling (Han et al., 2023). For instance, *NAC23* regulates sugar homeostasis, which in turn affects grain yield (Li et al., 2022). Furthermore, NAC127 and NAC129 form a complex that regulates sugar transport during rice grain filling (Ren et al., 2021), while the NAC24-NAP complex is known to regulate starch biosynthesis (Jin et al., 2023). NAC20 and NAC26 play redundant roles in regulating both starch and storage protein biosynthesis (Wang et al., 2020), and in activating albumin biosynthesis through interactions with RPBF (Wu et al., 2023). Similarly, in maize endosperm, *NAC128* and *NAC130* redundantly regulate the accumulations of starch and storage proteins (Zhang et al., 2019; Chen et al., 2023), and in wheat, *NAC019*, *NAC100* and *NAC-A18* are implicated in the regulation of storage proteins and starch during grain filling (Liu et al., 2020; Li et al., 2021; Wang et al., 2023). Collectively, these findings suggest that the transcriptional regulation of rice grain filling is a complex process that may act through multiple transcription factors and in multiple pathways.

In this study, we aimed to provide further understanding of rice grain filling through the identifications of other NAC family members involved in its regulation. Given that the rice NAC family was identified more than a decade ago, and with recent updates to rice genome annotation (Fang et al., 2008; Nuruzzaman et al., 2010), we accessed the expression profiles of NAC genes across various tissues from public database, and *NAC25* was selected for knockout analyses due to its specific expression in the developing rice endosperm. Our results revealed that loss-of-function of *NAC25*, the *nac25* mutants obtained showed chalky endosperm with an aberrant starch accumulation and a delayed degeneration of cytoplasmic membrane integrity in the starchy endosperm. Consistent with these observations, the expressions of genes related to the starch synthesis and cytoplasmic membranes degeneration were significantly down-regulated in *nac25* mutants. Furthermore, we found that NAC25 interacted with MADS29, a transcription factor previously implicated in rice grain filling and PCD (Yin and Xue, 2012; Yang et al., 2012).

## 2 Materials and methods

### 2.1 Plant materials and growth conditions

Rice plants (*Oryza sativa* L. *ssp*. *Japonica*), specifically cultivar Zhonghua 11 (ZH11, the wild type) and its derived mutants were cultivated either in experimental fields at either the Institute of Crop Sciences, Chinese Academy of Agricultural Sciences, or at the Institute of Botany, Chinese Academy of Sciences, Beijing. Alternatively, during the non-season periods, these plants were grown in growth chambers, which were maintained at a humidity level of 60% - 80%, with a 12 - hour light per day cycle. The day temperature was kept at 30 ± 2°C, while a night temperature was maintained at 22 ± 2°C.

### 2.2 Bioinformatics analyses

By querying the NAM domain (HMM: accession PF02365) against the Rice Genome Annotation Project Database (RGAP, https://rice.uga.edu/index.shtml) and RAP-DB Database (https://rapdb.dna.affrc.go.jp/index.html), we retrieved only one transcript of NAC genes. The phylogenetic tree for these NAC genes was constructed by using neighbor-joining method in MEGA 7.0, with 1,000 bootstrap replicates. Expression data of these NAC genes were obtained from RiceXPro database (Sato et al., 2013). Specifically, we utilized the RXP_0001 dataset, which provides spatio-temporal gene expressions for various tissues and organs throughout the entire growth period in the field. In addition, protein interactions were predicted using STRING (https://cn.string-db.org/cgi/input?sessionId=bhLchrlytDbK&input_page_show_search=on).

### 2.3 Quantitative real-time PCR

Total RNA was extracted using TRIzol™ reagent (15596018, Invitrogen), cDNA was synthesized using the PrimeScript™ RT reagent kit (Takara, Japan), and qRT-PCR was conducted using TB Green^®^ Premix Ex Taq™ II (Takara, Japan) on LightCycler^®^ 96 (Roche Life Science, Switzerland). Primers for qRT-PCR were designed using Primer3 (https://primer3.ut.ee) based on coding sequences of genes. The 2^-△△CT^ method was used to calculate relative expression levels with rice *Ubiquitin* gene as an internal control.

### 2.4 Genetic transformation and gene editing

To generate the *pNAC25::GUS* transgenic line, the *NAC25* promoter (2,000-bp upstream of the start codon) was cloned into the *pCAMBIA-1301* vector that carries a *β-glucuronidase* (*GUS*) reporter gene. To generate knockout mutants for *NAC25*, CRISPR/Cas9-based gene editing was performed as described (Ma et al., 2015). These constructs were transformed to ZH11 using *Agrobacterium*-mediated transformation (Hiei et al., 1994). Primers used are listed in **Supplementary Table S2**.

### 2.5 GUS assay

The GUS assay was performed as described (Fiers et al., 2004) and photographs were captured under a microscope (SMZ800N, Nikon).

### 2.6 Analyses of total starch and storage proteins

Total starch and storage proteins were extracted from mature caryopses, and their contents were measured as reported (López-Delgado et al., 2005; Liu et al., 2025a; Chen et al., 2018; Takemoto et al., 2002). Storage protein profiles were analyzed by SDS-PAGE as described.

### 2.7 Cell biological analyses

Rice caryopses at different developing stages were sectioned transversely and fixed in a modified FAA solution, vacuum-infiltrated for 30 minutes, prior to storage at 4°C overnight (Wu et al., 2016). The fixed samples were then dehydrated with an ethanol series, embedded in paraffin, sectioned with a microtome, stained with periodic acid-Schiff (PAS) (Wu et al., 2016), and imaged with a light microscope (Y-TV55, Nikon). Mature caryopses were dried at 37°C for one week before cracked, coated with gold, and examined under a scanning electron microscope (SEM, S-4800, Hitachi). Developing caryopses of ZH11 and *nac25* were collected at different DAPs, stained with Evans blue as described (Wu et al., 2016), and photographed using a microscope (SMZ800N, Nikon). The percentage of the stained area within the endosperm was quantitatively analyzed using ImageJ software (v1.59g; National Institutes of Health). Specifically, the endosperm area was manually delineated to exclude the outer caryopsis tissues. Subsequently, the threshold was adjusted to discriminate the positive staining areas. The percentage of stained area was calculated as the ratio between the threshold-defined area of positive staining and the total endosperm area.

### 2.8 Y2H, BiFC and LCI analyses

Yeast two-hybrid (Y2H) analysis was performed using the Matchmaker Gold Yeast Two-Hybrid System (Clontech) according to manufacturer’s instruction using the bait (*NAC25-pGBKT7*) and prey (*MADS29-pGADT7*) constructs. For BiFC assay, coding sequences of either *NAC25* or *MADS29* were in-frame fused to cYFP- and nYFP-fusion constructs (He et al., 2018), respectively, and transformed to rice protoplasts for transient expression. After 12 hours of culturing in dark, the fluorescence signal was detected using a confocal microscope (FV1000MPE, Olympus). Additionally, *NAC25* and *MADS29* were also ligated into the cLUC- and nLUC-fusion construct vectors, respectively, and transformed to *Agrobacterium* strain (EHA105), and then co-infiltrated pair-wisely into *Nicotiana benthamiana* leaves as reported (Chen et al., 2008). Luciferase signal was detected under a low-light cooled CCD camera (Tanon) after 2 day’s incubation. Primers used are listed in **Supplementary Table S2**.

### 2.9 Transcriptome analysis

RNA sequencing (RNA-seq) was performed on the Illumina Hiseq™ platform using endosperms collected at 9 DAP from ZH11 and *nac25-1* (three biological replicates each) to explore differentially expressed genes (DEGs). All reads were mapped to the rice reference genome (https://rice.uga.edu/index.shtml). Genes with *q*-value ≤ 0.05 and |FoldChange| ≥ 2 were identified as DEGs. Gene Ontology (GO) (http://www.geneontology.org/) and KEGG enrichment analyses (https://www.genome.jp/kegg/) were performed for DEGs.

## 3 Results

### 3.1 Phylogenetic and expression analyses of NACs in rice

To identify NAC transcription factors associated with rice endosperm development, we conducted comprehensive analyses on rice NAC family members using the Hidden Markov Model (HMM) of the NAM domain. By removing redundant or alternatively spliced transcripts of the same gene, we identified 144 putative NACs containing the NAM domain, including eight members found only in RGAP database and three members only in RAP-DB database (**Supplementary Table S1**). Constructed phylogenetic tree classified NAC genes into three groups, with the group C containing CUC, SWN and NTL (**Supplementary Figure S1**). Subsequently, we searched for NAC members that are specifically or highly expressed in endosperm using the Rice Expression Profile (RiceXPro) database (http://ricexpro.dna.affrc.go.jp/). Among 93 NAC members incorporated, *NAC25*, *NAC23*, *NAC26*, *NAC20*, *NAC24*, *NAC128* and *NAC127* formed a distinct clade, exhibiting high expression levels in the endosperm (**Supplementary Figure S2**). Of these, *NAC23*, *NAC26*, *NAC20*, *NAC24* and *NAC127* have been previously reported for their specific expression patterns. Interestingly, *NAC128* as a highly expressed in caryopses, comprised only 161 amino acids, lacking the C-terminal regulatory region when compared with other members (**Supplementary Figures S3A, B**). Based on these findings, we focused on the less studied *NAC25* for further investigation to explore its role in rice grain filling.

### 3.2 *NAC25* was specifically expressed in the rice endosperm

**Figure 1:**
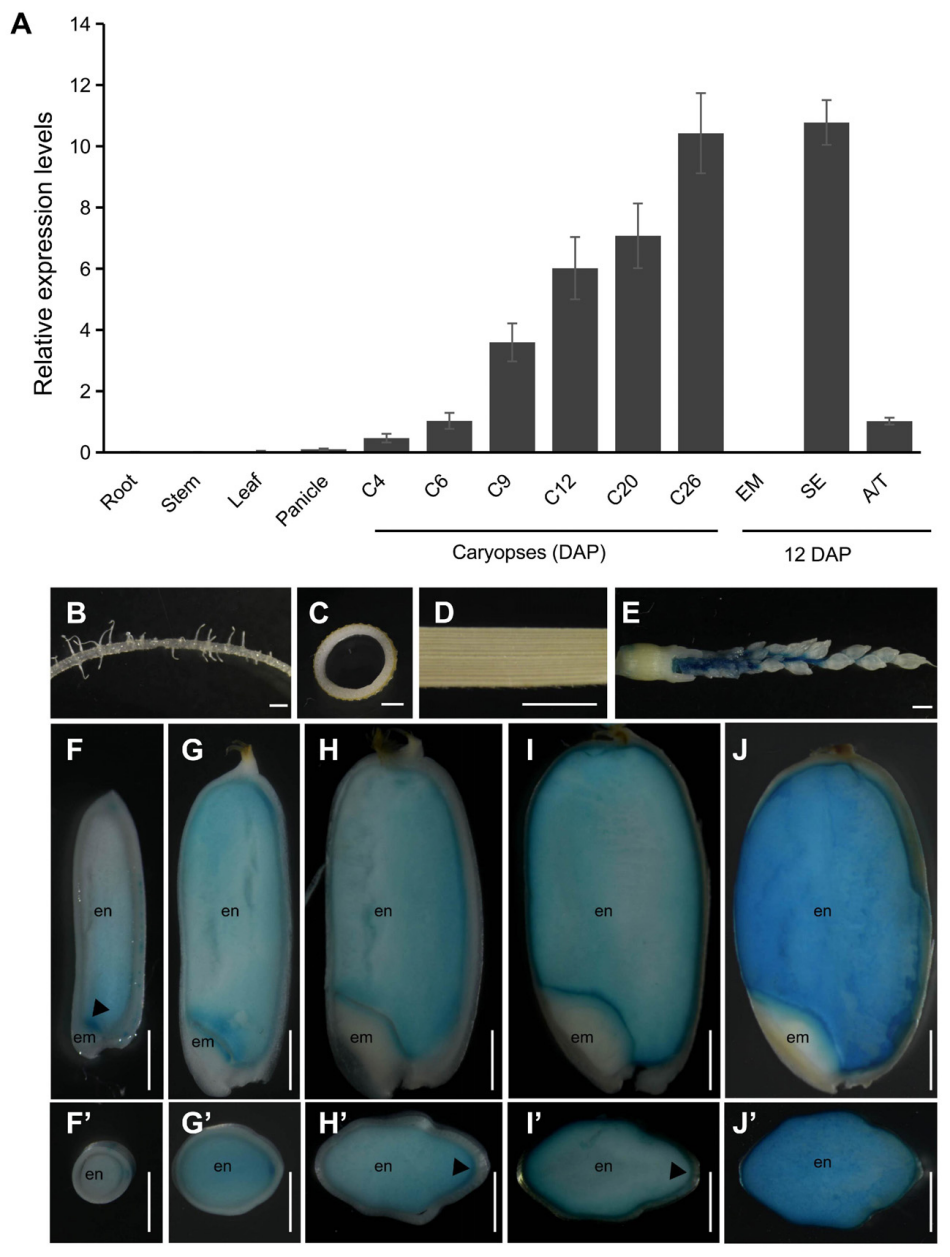
Expression analysis of *NAC25*. **(A)** qRT-PCR analysis showed increased expressions of *NAC25* in developing caryopses at 4 to 26 DAP (C4 to C26), and the expression was specific in starchy endosperm (SE), not in embryo (EM), collected at 12 DAP. DAP, days after pollination. A/T, a mixed aleurone and testa sample collected at 12 DAP. (B-J’) GUS assays performed in transgenic plants carrying *pNAC25::GUS*, to show the GUS expressions in inflorescence stem (**E**, panicles of 3.5 cm long) and endosperm (en). Note no expression in roots **(B)**, stems **(C)**, leaves **(D)** and embryos (em). Caryopses collected at 4, 6, 9, 15, 25 DAP, and sectioned either longitudinally **(F-J)** or transversally **(F’-J’)**. Scale bars, 1 mm.

To investigate the function of *NAC25*, we first conducted qRT-PCR to examine its expression patterns in ZH11. Our results indicated that *NAC25* was specifically expressed in developing caryopses, and was undetectable in tissues such as roots, stems and leaves (**Figure 1A**). During caryopsis development, increased *NAC25* expression was observed from 4 to 26 DAP, with expression levels continuously increasing and peaking at 26 DAP. Within the caryopsis, the *NAC25* expression was detected in the starchy endosperm, mixed aleurone and testa sample, while no expression was detected in the embryo.

To define the *NAC25* expression in detail, transgenic plants carrying a *pNAC25::GUS* reporter construct were generated, and GUS assay was performed across various tissues. Results showed that no GUS signal was observed in the roots (**Figure 1B**), stems (**Figure 1C**), leaves (**Figure 1D**) and embryos (**Figures 1F–J**). In contrast, it was detected in inflorescence stems before flowering (**Figure 1E**), and highly expressed in the starchy endosperm after fertilization (**Figures 1F–J**). During the endosperm development, the *GUS* expression was strongly observed in embryo-surrounding endosperms at 4 DAP (**Figure 1F**, indicated by an arrowhead), and expanded to the entire starchy endosperm from 6 DAP onwards (**Figures 1G-J**). In the transversal sections, elevated expression was noted in the dorsal aleurone, adjacent to nucellar projection, at 9 and 15 DAP (**Figures 1H, I**, indicated by arrowheads), two stages known to be active in grain filling (Wu et al., 2016).

### 3.3 Mutations of *NAC25* led to defective grain filling

**Figure 2:**
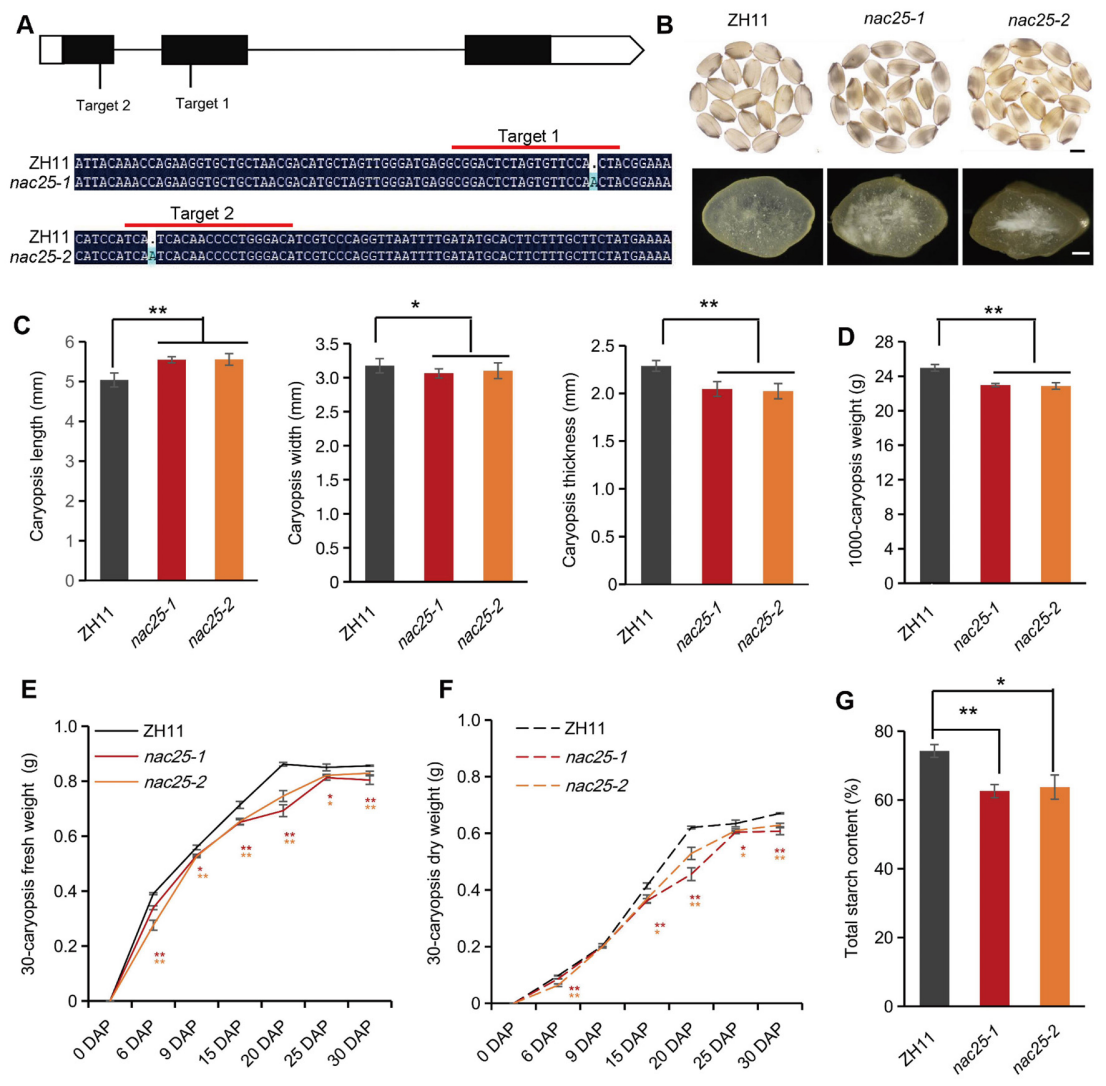
Mutations of *NAC25* led to defective grain filling. **(A)** *nac25-1* and *nac25-2* knockout mutants generated by CRISPR/Cas9. Upper panel, a gene model of *NAC25* to show positions of single-nucleotide insertions in *nac25-1* and *nac25-2*. Lower panel, mutation sites in *nac25-1* and *nac25-2*, as compared with ZH11 sequences. The sequences under the red lines are the target sequences. **(B)** Mature caryopses of *nac25-1* and *nac25-2* showed chalky endosperm phenotype as compared to the semi-transparent endosperm in ZH11. Upper panel, side views of intact caryopses, scale bars, 2 mm; lower panel, surface views of cracked caryopses, scale bars, 500 μm. **(C)** Mature *nac25* caryopses showed significant reductions in both width and thickness, while showing an increase in length. **(D)** The 1,000-caryopsis weights of *nac25* mutants were reduced compared with ZH11. Data are shown as means ± SD (**: P < 0.01, *: 0.01< P < 0.05, based on Student’s t-test). **(E, F)** Changes of the fresh and dry weights of 30-caryopsis at 6, 9, 15, 20, 25 and 30 DAP from ZH11 and *nac25* mutants. Note the grain filling in *nac25* mutants were slow and the final weights were less than ZH11. Data are shown as means ± SD (*: 0.01 < P < 0.05 and **: P < 0.01, based on Student’s t-test). **(G)** Total starch content in mature caryopses showed significantly reduced accumulation in *nac25* as compared to that in the ZH11 caryopses. Data are shown as means ± SD (*: 0.01 < P < 0.05 and **: P < 0.01, based on Student’s t - test).

To further elucidate the function of *NAC25*, we generated *nac25* mutants by CRISPR/Cas9-based gene editing in ZH11. Two guide RNAs were designed to target the first and the second exons of *NAC25*, and two independent knockout lines, *nac25-1* and *nac25-2*, were obtained (**Figure 2A**). Sequencing analysis showed that both of them carried a 1-bp insertion in the target sites, leading to frame-shifts and consequently truncated proteins as expected (**Figure 2A**; **Supplementary Figures S4A–C**). Compared with ZH11, mature caryopses of *nac25-1 and nac25-2* exhibited chalky appearance (**Figure 2B**), but no detectable differences in plant architecture and panicle morphology (**Supplementary Figure S4D, F-H**). The 1,000-caryopsis weights of these *nac25* mutants were reduced to 92% of that of ZH11 (**Figure 2D**), which was primarily caused by significant reductions in both width and thickness of these *nac25* caryopses (**Figure 2C**; **Supplementary Figure S4E**). The fresh and dry caryopses weights were measured throughout the caryopsis development, and results showed that the grain filling in *nac25* mutants were slow and the final caryopsis weights were less when compared with those of ZH11 (**Figures 2E, F**). We also analyzed main storage products in them, and results showed that the total starch content in *nac25* mutants was decreased from 74.3% in ZH11 to 62.6% in *nac25-1* and 63.7% in *nac25-2* (**Figure 2G**). No evident differences were observed in storage proteins, as assessed using SDS-PAGE (**Supplementary Figure S4I**). These findings suggest that *NAC25* plays an important role in grain filling and starch biosynthesis in rice endosperm.

### 3.4 Aberrant starch accumulation in *nac25* endosperms

**Figure 3:**
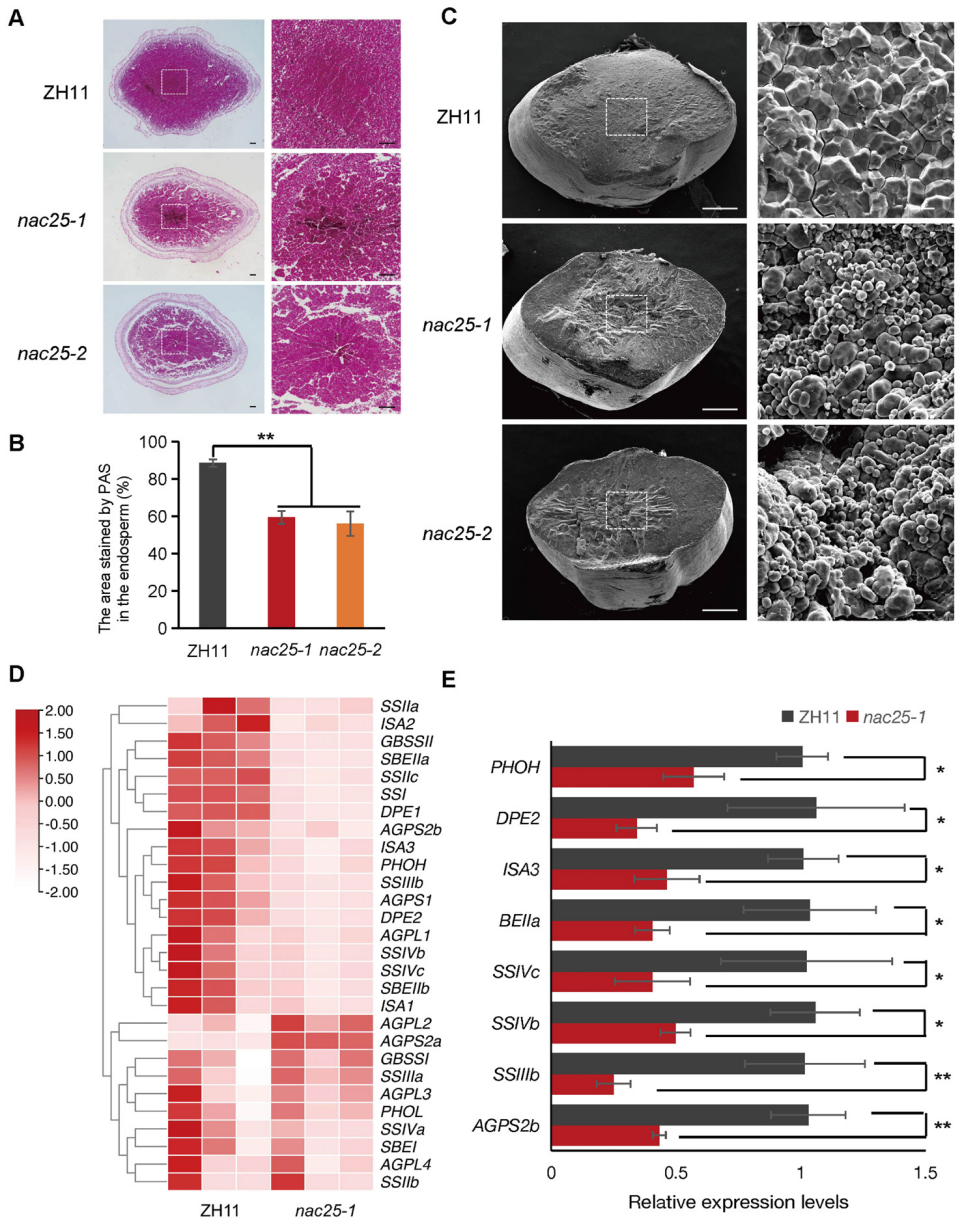
Aberrant starch accumulation in *nac25* endosperms. **(A)** Light microscopy observation of periodic acid-Schiff (PAS) stained cross-sectioned caryopses collected at 15 DAP. Conspicuous air spaces were observed in the central region of *nac25* caryopses, whereas the corresponding area in ZH11 was densely packed with starch grains. The right panel is the central region of left one. Scale bars, 100 μm. **(B)** The quantification results of the stained area in the endosperm of (**Figure 3A**) showed that the areas with starch accumulation in the endosperms of *nac25* mutants were less compared to ZH11. Data are shown as means ± SD (**: P < 0.01, based on Student’s t-test). **(C)** Scanning electron microscopy observation of the starchy endosperm of ZH11 and *nac25* mutants showed loosely packed starch grains in *nac25*, as compared to tightly packed ones in ZH11. The right panel is the central region of left one. Scale bars, 500 μm (left panel) and 10 μm (right panel). **(D)** Heatmap to show the relative expression levels of starch biosynthetic genes in the endosperm of ZH11 and *nac25-1* at 9 DAP based on RNA-seq data. **(E)** qRT-PCR analysis showed expression levels of starch biosynthesis-related genes were significantly downregulated in the *nac25-1* endosperm compared with ZH11. Data are shown as means ± SD (*: 0.01 < P < 0.05 and **: P < 0.01, based on Student’s t-test).

Light and scanning electron microscopy (SEM) were used to examine cross-sectioned *nac25* caryopses, collected at the 15 DAP and mature stage, respectively, to decipher their possible defects. At 15 DAP, the central region of the ZH11 endosperm was mostly filled with starch grains that stained red by PAS (Wu et al., 2016), while conspicuous air spaces were observed in the same region of the *nac25* endosperms (**Figure 3A**). Besides, areas stained by PAS in the endosperms of *nac25* mutants were much less when compared to those in ZH11, suggesting a slow and compromised starch accumulation in the mutants (**Figure 3B**). As expected, at the mature stage, SEM analysis revealed that starch grains in the *nac25* endosperms were chalky and loosely packed, in contrast to those densely packed compound starch grains in those of ZH11 (**Figure 3C**). RNA sequencing (RNA-seq) was then performed in endosperms excised from *nac25-1* and ZH11 at 9 DAP. A total of 4,242 differentially expressed genes (DEGs) were identified, including 3,100 down-regulated and 1,142 up-regulated ones. Among those DEGs, genes involved in ‘carbohydrate metabolism’ were enriched (**Supplementary Figure S5A**), which is evident in starch biosynthesis pathway (**Figure 3D**). qRT-PCR analyses showed that, compared with ZH11, expression levels of starch synthesis-related genes, including *AGPS2b*, *SSIIIb*, *SSIVb*, *SSIVc*, *BEIIa*, *ISA3*, *DPE2* and *PHOH*, were significantly down-regulated in the *nac25-1* endosperm, which confirms the results observed in RNA-seq (**Figure 3E**). These results together suggest a role of *NAC25* in starch biosynthesis.

### 3.5 Compromised degeneration of cytoplasmic membrane integrity in *nac25* endosperms

**Figure 4:**
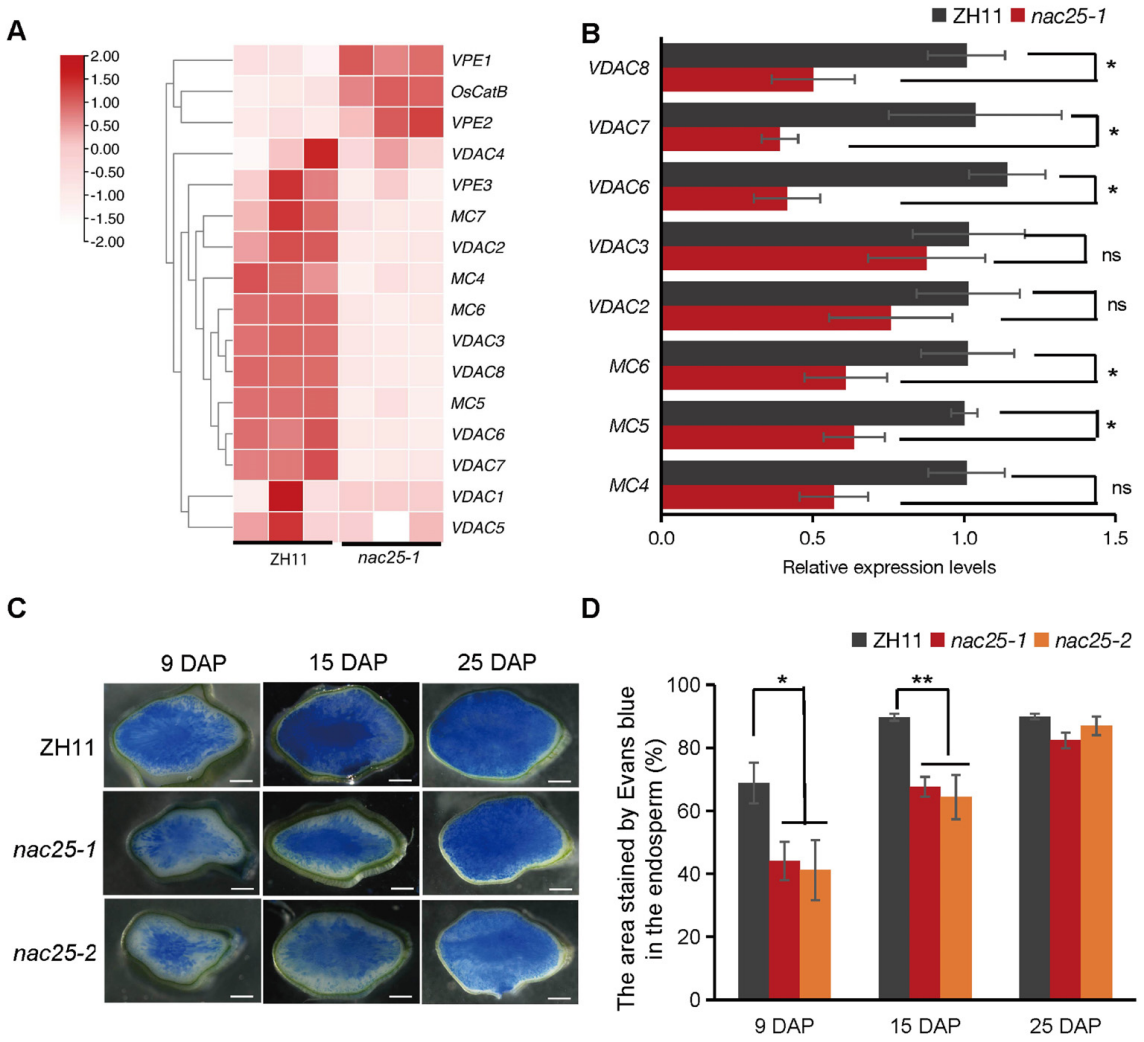
Compromised degeneration of cytoplasmic membrane integrity in *nac25* endosperms. **(A)** Heatmap showing the relative expression levels of genes related to metacapases (MC) and voltage-dependent anion channels (VDAC) in the endosperm of ZH11 and *nac25-1* at 9 DAP from RNA-seq data. **(B)** Validation by qRT-PCR showed that the expression levels of *MC4*, *MC5* and *MC6*, as well as *VDAC6*, *VDAC7* and *VDAC8* were significantly downregulated in *nac25-1* endosperm at 9 DAP. Data are shown as means ± SD (*: 0.01 < P < 0.05 and **: P < 0.01, based on Student’s t-test; ns: P>0.05, not significant). **(C)** Evans Blue staining of caryopses of ZH11 and *nac25* mutants at 9 DAP, 15 DAP, and 25 DAP, showing the degeneration of the cytoplasmic membrane integrity were much slower in *nac25* at both 9 and 15 DAP. Colored in blue indicate tissues with degenerated cytoplasmic membrane integrity. Scale bars, 500 μm. **(D)** The quantification results of the stained area by Evans blue in the endosperm of **Figure 4C** showed that the areas with degenerated cytoplasmic membrane integrity in the endosperms of *nac25* mutants were significantly smaller than those in ZH11 at 9 DAP and 15 DAP. Data are shown as means ± SD (*: 0.01 < P < 0.05 and **: P < 0.01, based on Student’s t-test).

During rice grain filling, the cytoplasmic membrane integrity is gradually lost in the starchy endosperm, coinciding with the rapid starch accumulation in the tissue (Wu et al., 2016; Liu et al., 2022a). Apart from genes involved in ‘carbohydrate metabolism’, we observed that genes involved ‘cell growth and death’, ‘cytoplasm’ and ‘cytoplasmic part’ were also enriched in DEGs (**Supplementary Figures S5A, B**). As showed in **Figure 4A**, genes related to metacapases (MC) and voltage-dependent anion channels (VDAC) from RNA-seq data were indeed down-regulated. As validated by qRT-PCR, expression levels of *MC4*, *MC5* and *MC6*, as well as *VDAC6*, *VDAC7* and *VDAC8* were significantly down-regulated in *nac25-1* (**Figure 4B**). Evans blue, which stains tissues with degenerated cytoplasmic membrane integrity in blue (Wu et al., 2016), showed that areas with degenerated cytoplasmic membrane integrity were gradually expanded from the central to the outer regions of the starchy endosperm in both ZH11 and *nac25* mutants (**Figure 4C**). In the endosperms of *nac25* mutants, the proportions of the areas with degenerated cytoplasmic membrane integrity were 59.89% to 64.06%, and 71.74% to 75.40% of those in ZH11 at 9 and 15 DAP, respectively, indicating that the degeneration of the cytoplasmic membrane integrity were slower in *nac25* at these stages (**Figures 4C, D**). At 25 DAP, cells with degenerated cytoplasmic membranes had expanded throughout the entire starchy endosperm in both ZH11 and *nac25* mutants, and no significant variation was observed (**Figures 4C, D**). These results indicate that *NAC25* regulated the degeneration of cytoplasmic membrane integrity in the starchy endosperm.

### 3.6 NAC25 interacted with MADS29

**Figure 5:**
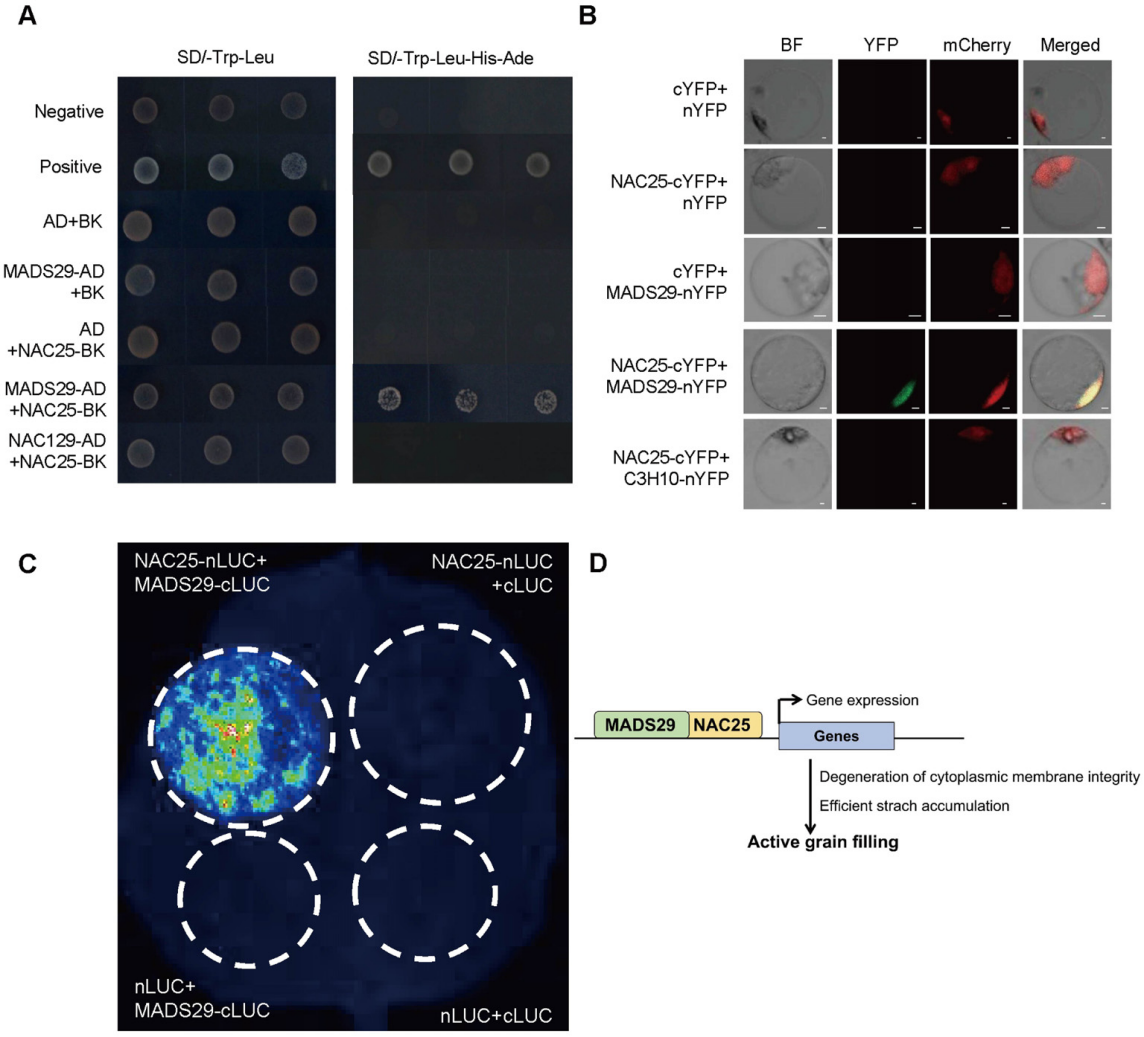
NAC25 interacted with MADS29. **(A)** Yeast two-hybrid assay showed the interaction between NAC25 and MADS29. Yeast cells transformed with pGBKT7-53 and pGADT7-T were used as the positive control, while pGBKT7-Lam and pGADT7-T were used as the negative control. **(B)** Bimolecular fluorescence complementation (BiFC) analysis revealed the interaction between NAC25 and MADS29. OsH2B-mCherry was used as a nuclear marker. Scale bars, 2 μm. **(C)** Luciferase complementation imaging (LCI) assay confirmed that NAC25 interacted with MADS29. **(D)** A proposed model for the role of NAC25 in rice grain filling: Transcription factor NAC25 (in orange) interacts with MADS29 (in green) to activate expressions of genes related to both degeneration of cytoplasmic membrane integrity and starch biosynthesis in the endosperm, consequently promoting the grain filling.

To further investigate the role of *NAC25* in starch biosynthesis and the degeneration of cytoplasmic membrane integrity during rice grain filling, we first used the STRING database to predict potential protein-protein interactions. Based on this analysis, MADS29 was identified as a potential interactor for NAC25 (**Supplementary Figure S6**). MADS29 has been previously reported to be expressed in rice caryopses, and implicated in grain filling primarily through the regulation of programmed cell death (PCD) of both maternal and filial tissues (Yin and Xue, 2012; Yang et al., 2012). To validate the interaction, we performed several assays including yeast two-hybrid (Y2H), bimolecular fluorescence complementation (BiFC), and luciferase complementation imaging (LCI). NAC129, an unrelated transcription factor (Ren et al., 2021), was used as a negative control, yeast cells co-transformed with *MADS29-AD* and *NAC25-BK* constructs grew well on selective medium, confirming the interaction between these proteins (**Figure 5A**). In the BiFC assay, the unrelated transcription factor C3H10 (a CCCH-Zinc Finger) was used as a negative control (Seong et al., 2020), and we detected yellow fluorescent protein (YFP) signal in nuclei of rice protoplasts when co-transformed with *NAC25-cYFP* and *MADS29-nYFP*, while no YFP signal was observed when transformed with *NAC25-cYFP* and *C3H10-nYFP* (**Figure 5B**). LCI assay showed positive fluorescence signal in tobacco leaves when co-infiltrated with *NAC25-nLUC* and *MADS29-cLUC* constructs (**Figure 5C**). These results together confirmed the physical interaction between NAC25 and MADS29.

## 4 Discussion

In this study, we identified a member of NAC gene family, *NAC25*, that is expressed specifically in the rice endosperm, and characterized two *nac25* mutant alleles obtained through gene editing. We showed that mutations of *NAC25* led to chalky endosperm with reduced grain weights. We further showed that these phenotypes were caused by compromised grain filling and delayed degeneration of cytoplasmic membrane integrity. Biochemical analyses demonstrated that NAC25 interacted with MADS29, a MADS family transcription factor that has been showed to promote the PCD of maternal and filial tissues in rice caryopsis. These observations led us to hypothesize that *NAC25*, expressed in rice endosperm, may act together with *MADS29* to activate expressions of genes related to both starch biosynthesis and degeneration of cytoplasmic integrity in rice starchy endosperm, and consequently the grain filling (**Figure 5D**). Further studies of this regulatory module may help us to develop rice varieties with efficient grain filling, and higher yields.

Through in silico analyses, together with verification using qRT-PCR, we identified *NAC25* as a transcription factor predominantly expressed in developing endosperm (**Figure 1A**; **Supplementary Figure S2**), together with *NAC127*, *NAC128* and *NAC129* in the same phylogenetic tree (Fang et al., 2008). It has been showed before that mutations and over-expressions of either *NAC127* or *NAC129* resulted in slower grain filling when grown under heat stress condition, and the double mutant of *NAC127* and *NAC129* exhibited more severe phenotype than single mutants (Ren et al., 2021). Similarly, defective grain filling has also been observed when both *NAC20* and *NAC26* were mutated (Wang et al., 2020; Wu et al., 2023). Both *NAC20* and *NAC26* are expressed specifically in rice starchy endosperm, and the primary function of them are regulating albumin deposits (Wu et al., 2023). It has been shown recently that NAC25 and NAC20/26 form regulatory loops to regulate starch synthesis (Wang et al., 2024). In this study, we showed that *NAC25* is expressed specifically in the rice endosperm, and regulates both starch biosynthesis and cytoplasmic membrane integrity. These findings imply that multiple NAC family transcription factors are expressed specifically in the rice endosperm, and are involved in regulating different stages and different developmental events of storage product accumulations.

It is interesting to note that defects observed in *nac25* mutants are less pronounced than several rice grain filling mutants such as *flo* (Ren et al., 2023). This could be attributed to the redundant roles of *NAC25*, *NAC127*, *NAC128* and *NAC129* in the same phylogenetic clade, as all of them were expressed in developing caryopses and shared certain similarities in their NAM domains (**Supplementary Figures S3A, B**). Therefore, the generations of triple or quadruple mutants are necessary to fully elucidate the distinct and overlapping roles of these NAC transcription factors in grain filling.

During rice grain filling, different nutrients are accumulated in different tissues of the endosperm: lipids, soluble proteins and vitamins are in the outer aleurone layer, while starch and glutelin are in the inner starchy endosperm (Liu et al., 2022a). Although aleurone and starchy endosperm share the same developmental origin, they exhibit markedly different cell fates. In mature cereal endosperm, the aleurone layer remains a live tissue, whereas the starchy endosperm as the major source of white rice is a dead tissue, as the consequence of PCD (Liu et al., 2022a). During the rapid accumulation of starch grains in rice, starchy endosperm gradually loses the cytoplasmic membrane integrity, from the central region towards the periphery, to allow efficient sugar translocation in the starchy endosperm with shared cytoplasms, as proposed recently by Liu et al. (2022a). During this process, an increased activity of caspase-like protease has also been shown (Kobayashi et al., 2013). It has been reported before that suppressed *MADS29* expression impairs rice grain filling by delayed PCD of both the maternal and filial tissues (Yin and Xue, 2012; Yang et al., 2012). In this study, we showed that NAC25 interacted with MADS29 (**Figures 5A–C**), and expressions of genes of starch biosynthesis, MC and VDAC members were significantly reduced in *nac25-1* (**Figures 3E**, **4B**). These results imply that the delayed degeneration of cytoplasmic membrane integrity in *nac25* mutants may compromise the formation of a shared cytoplasm within the starchy endosperm, thus hindering efficient sugar transports and consequently starch biosynthesis. These results suggest that an orderly progression of the degeneration of cytoplasmic membrane integrity in the starchy endosperm is crucial for efficient grain filling in rice.

Previous studies have shown that transcription factors may form complexes to act on specific pathways during grain filling. *NF-YB1*, an aleurone specifically expressed NF-Y family transcription factor, plays a critical role in grain filling (Bai et al., 2016). Several transcription factors that interact with NF-YB1 exhibit similar phenotypes when mutated. In particular, NF-YC12 and MADS14 facilitate the nuclear import of NF-YB1 through heterodimerization, the interaction of NF-YB1 with NF-YC12, bHLH144, MADS14, MYB73 and MADS1 enhanced expressions of genes related to sucrose transport and starch synthesis (Bello et al., 2019; Xiong et al., 2019; Feng et al., 2022; Liu et al., 2024a, Liu et al., 2025b). Additionally, NF-YB1 also interacts with bZIP76, a regulator of early endosperm cellularization, and with ERF115 that is involved in ethylene signaling (Niu et al., 2020; Xu et al., 2016; Liu et al., 2022b). Moreover, NF-YB1 also showed to interact with MYB73 to jointly regulate the expression of *YUC11*, thereby inducing the auxin biosynthesis (Liu et al., 2024a). These findings suggest that *NF-YB1* may serve as a core regulator of grain filling through multiple pathways. Similarly, the mutations of *RPBF* led to a more severe phenotype in rice endosperm, with more opaque and wrinkled caryopses than mutations of its interacted proteins including RISBZ1/bZIP58, NAC20, NAC26, suggesting that RPBF may act as a core regulator in rice grain filling, especially the regulation of albumin through interactions with NAC20 and NAC26 (Wu et al., 2023; Yamamoto et al., 2006; Kawakatsu et al., 2009). In this study, we discovered that starch biosynthesis and the degeneration of cytoplasmic membrane integrity are compromised in rice starchy endosperm when *NAC25* was mutated (**Figures 2G**, **3A–C**, **4C, D**). The defect in grain filling of *nac25* mutants was not as pronounced as that in mutants of NAC25-interacting transcription factor MADS29. It is plausible that MADS29 acts as a core transcription factor in multiple pathways such as the degenerations of maternal and filial tissues, the storage compounds accumulation, and the hormone homeostasis during rice grain filling (Yin and Xue, 2012; Yang et al., 2012; Nayar et al., 2013), and NAC25 functions as a specific cofactor for MADS29 in regulating starch accumulation and degeneration of cytoplasmic integrity in the starchy endosperm. In wheat it has been observed that TaNF-YB1 acts as a cooperative auxiliary factor to TaMADS29, synergistically regulating the expression of genes related to endosperm cell death and grain filling (Liu et al., 2023b). Genetic analyses are needed in the future to confirm the speculated upstream-downstream relationship of NAC25 and MADS29.

## Data Availability

The data presented in the study are deposited in the NCBI Sequence Read Archive (SRA) repository, accession number PRJNA1227929.
