# Supplementary figures and images for: NAC25 transcription factor regulates the degeneration of cytoplasmic membrane integrity and starch biosynthesis in rice endosperm through interacting with MADS29
Source: Front Plant Sci. 2025 Mar 18;16:1563065. doi: 10.3389/fpls.2025.1563065 (PMC11958719; doi:10.3389/fpls.2025.1563065)

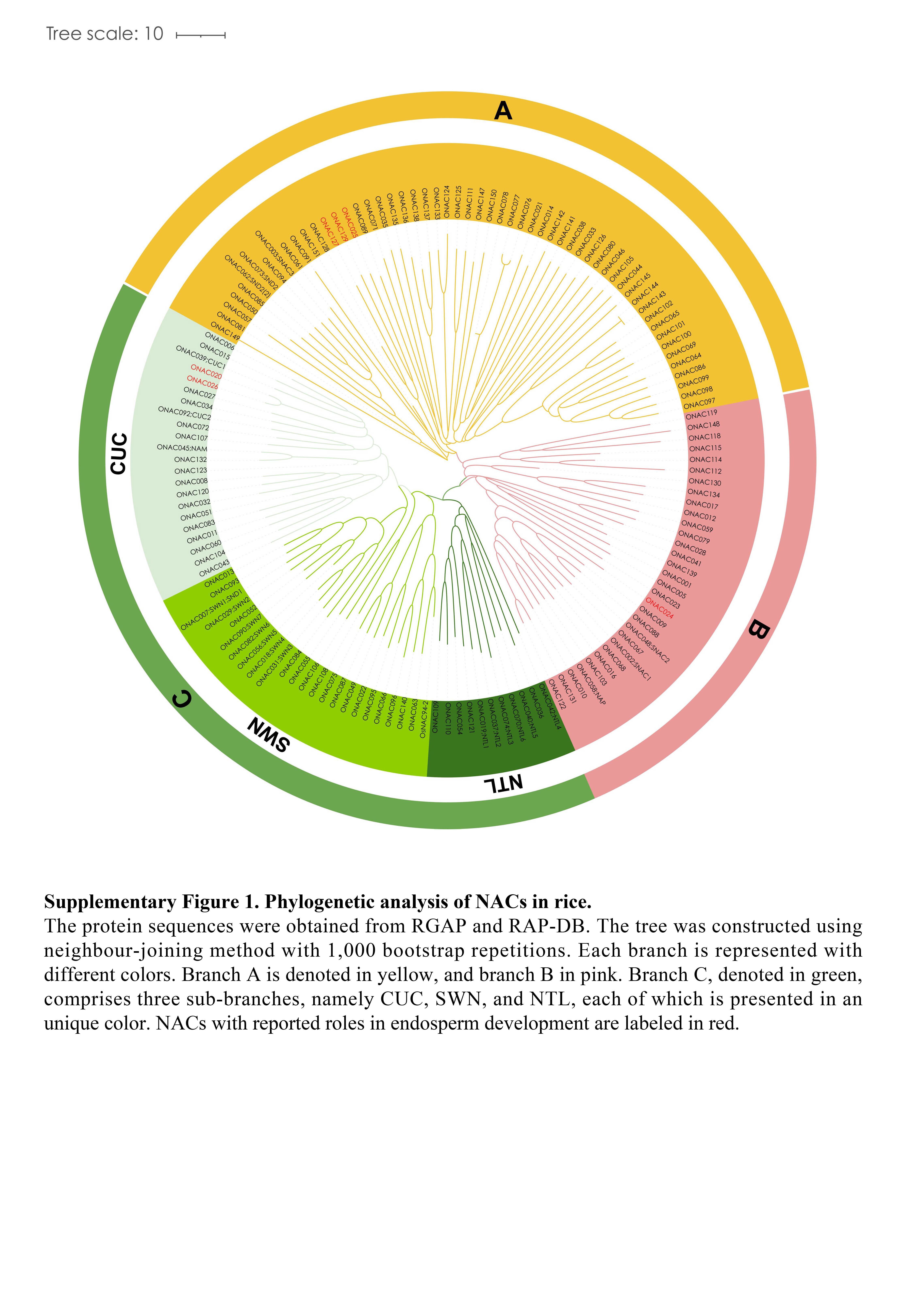

Supplement: Supplementary file 3 [file Image1.jpeg]

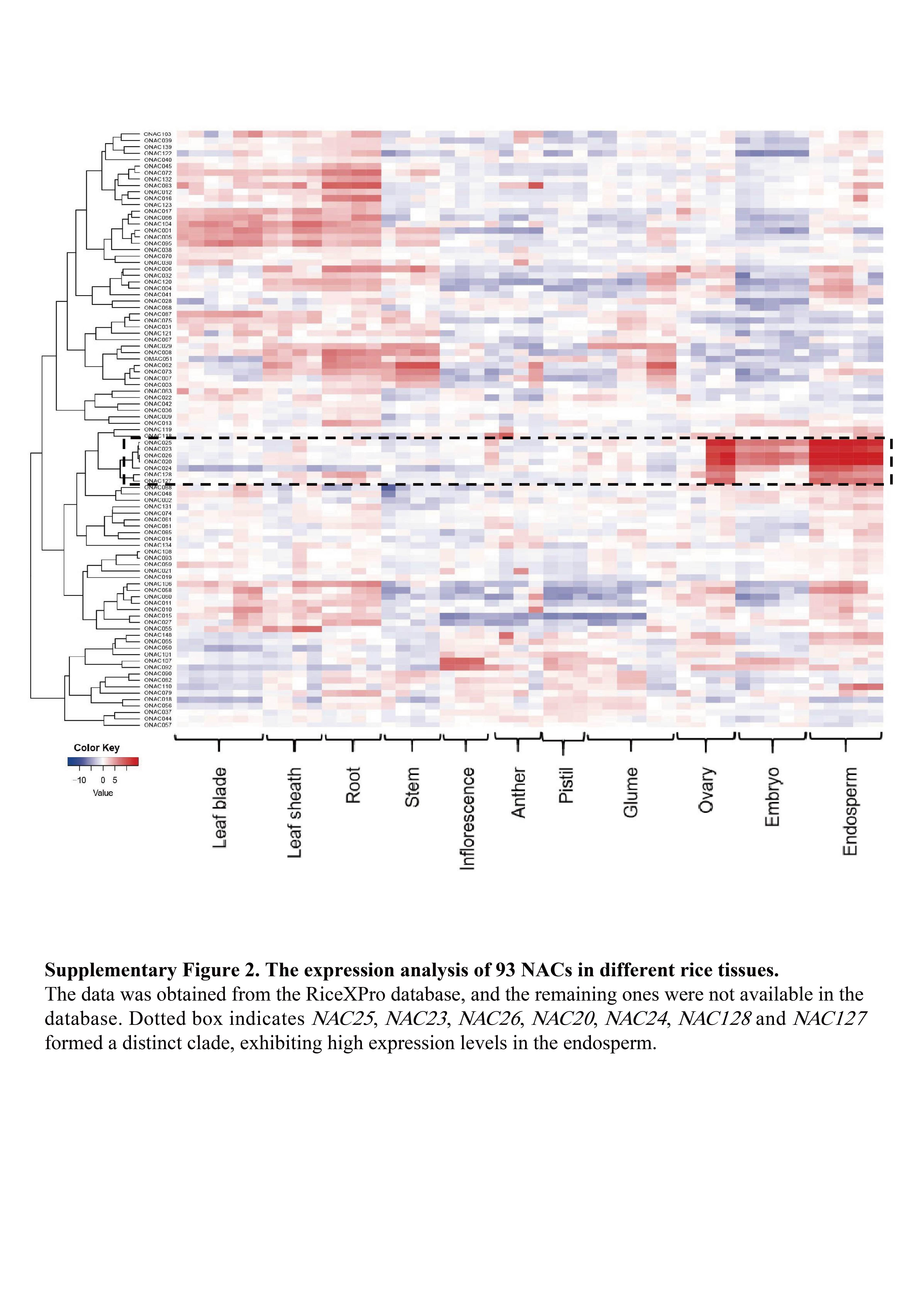

Supplement: Supplementary file 4 [file Image2.jpeg]

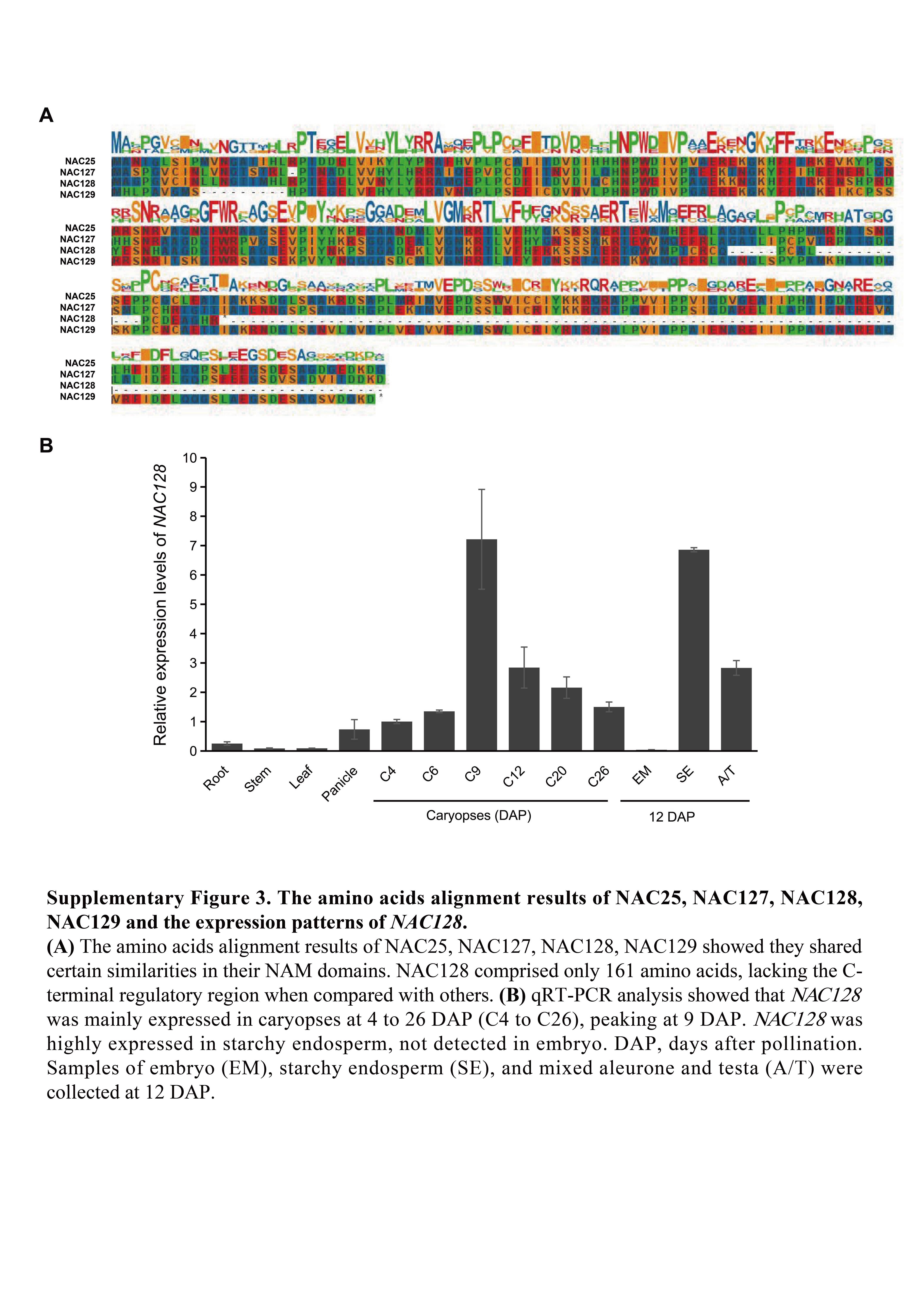

Supplement: Supplementary file 5 [file Image3.jpeg]

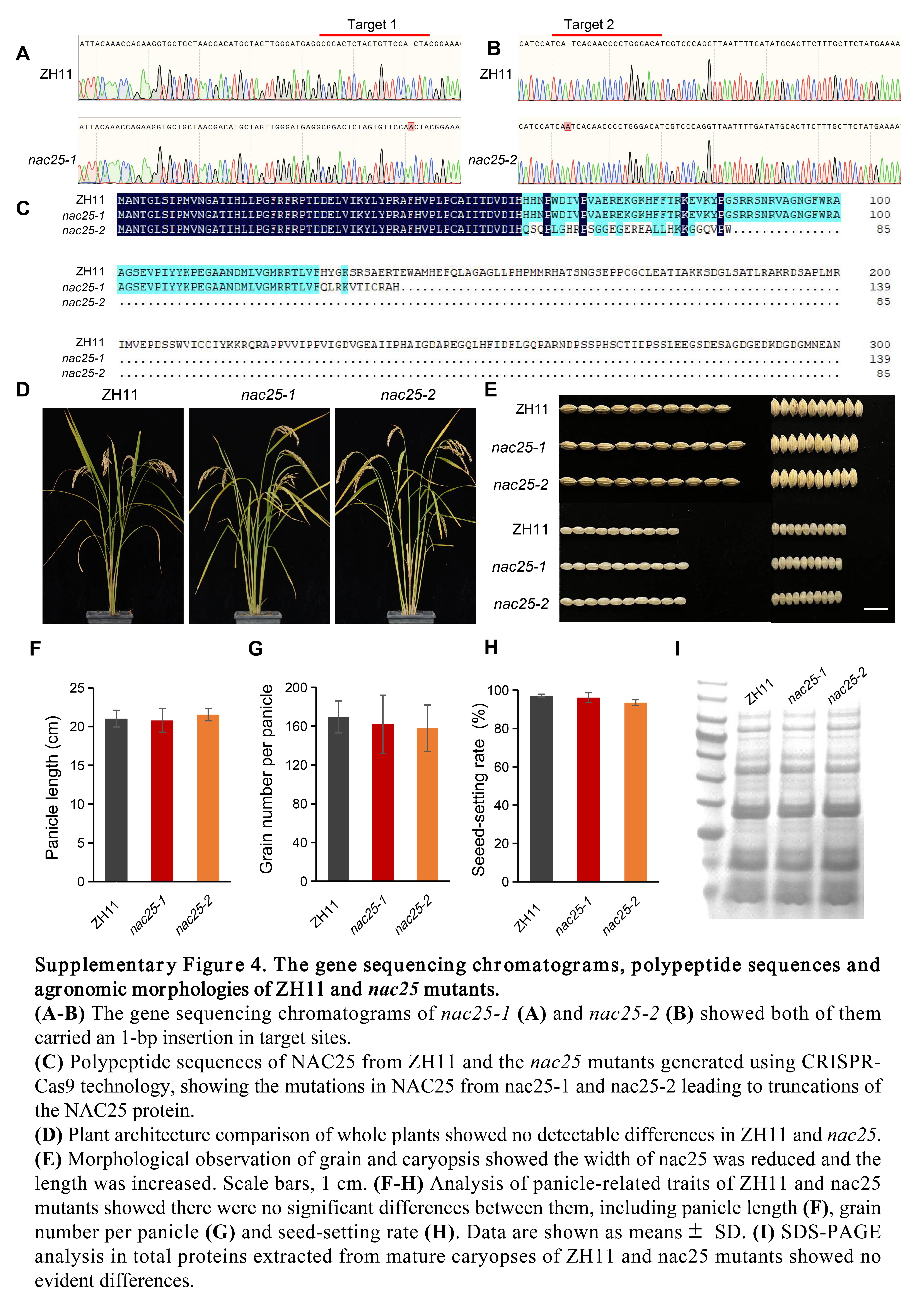

Supplement: Supplementary file 6 [file Image4.jpeg]

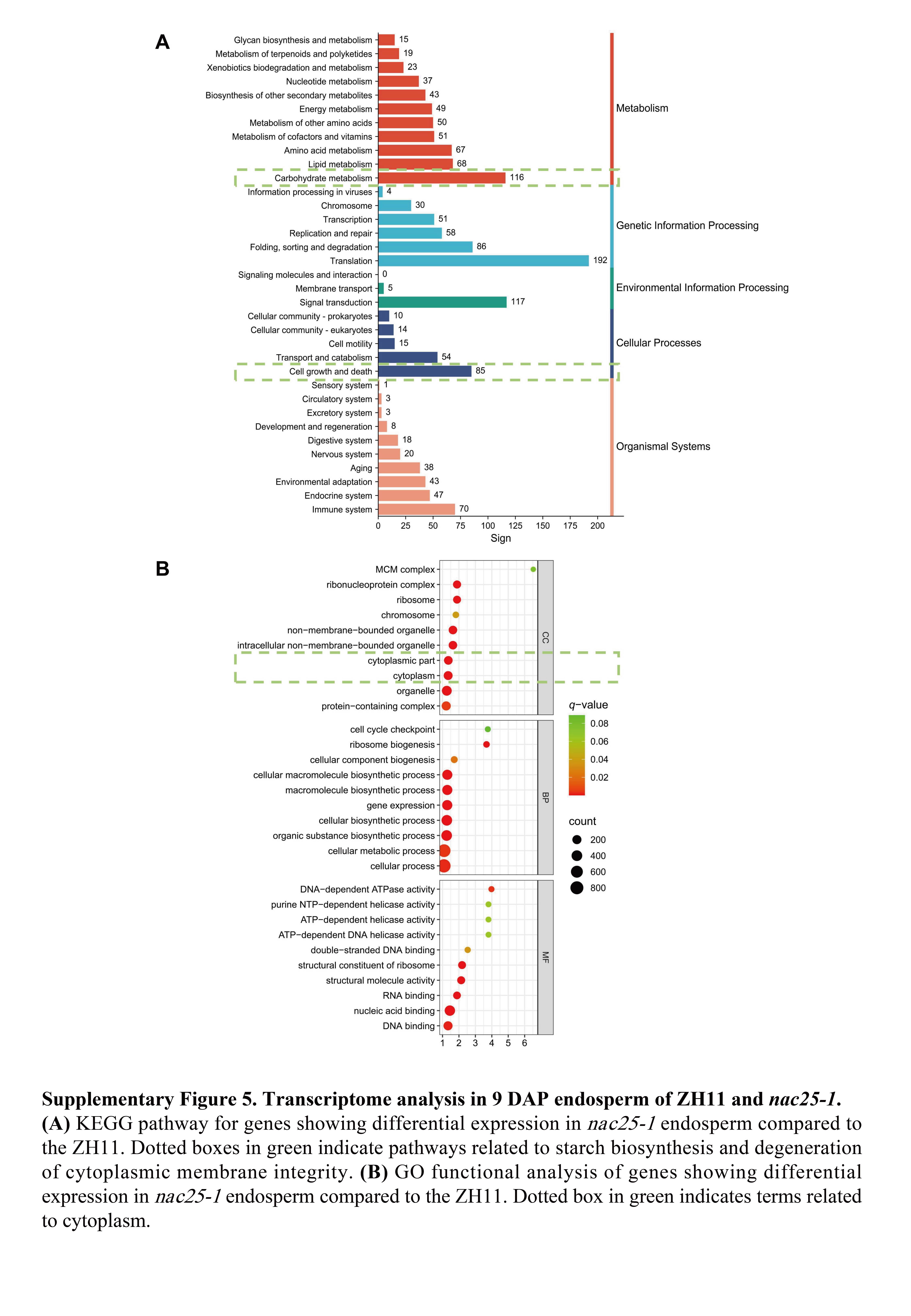

Supplement: Supplementary file 7 [file Image5.jpeg]

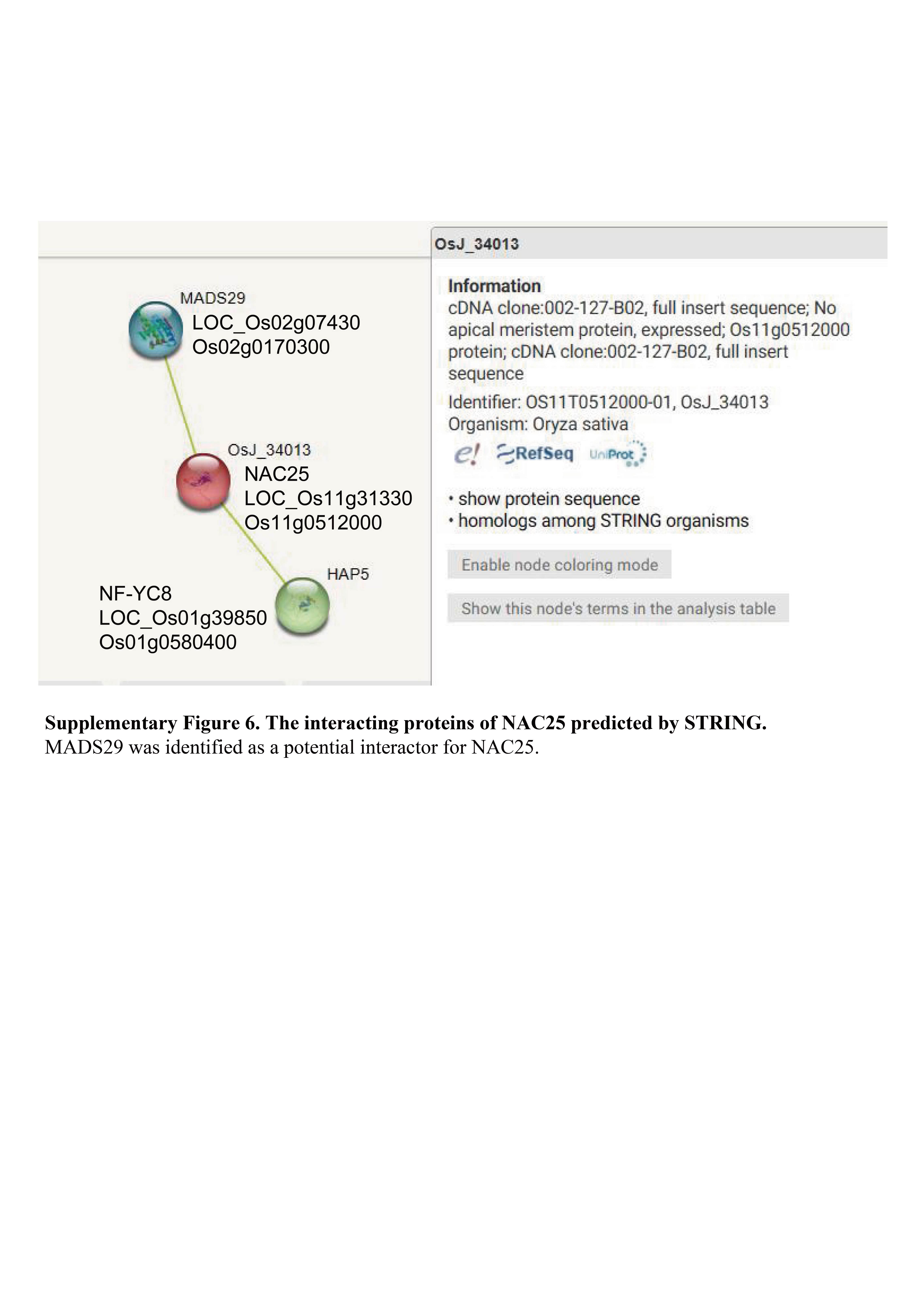

Supplement: Supplementary file 8 [file Image6.jpeg]
